# Supplementary material for: Space and Vine Cultivar Interact to Determine the Arbuscular Mycorrhizal Fungal Community Composition
Source: J Fungi (Basel). 2020 Nov 27;6(4):317. doi: 10.3390/jof6040317 (PMC7712214; doi:10.3390/jof6040317)
Supplement: Supplementary file 1 [file jof-06-00317-s001.zip › Suppl. material Fig. S1.pdf]

Supplementary Figure 1. Maximum Likelihood phylogenetic tree obtained with IQTREE (Nguyen et al., 2015) from a combined MAFFT (Katoh & Standley, 2013) alignment of the virtual taxa (VTX codes) found in the present study and available representative arbuscular mycorrhizal fungi sequences from GenBank (accession codes provided after species names). Bootstrap support values ≥ than 90 are shown. The sequence of the saprotrophic fungus *Mortierella polycephala* has been used to root the tree.

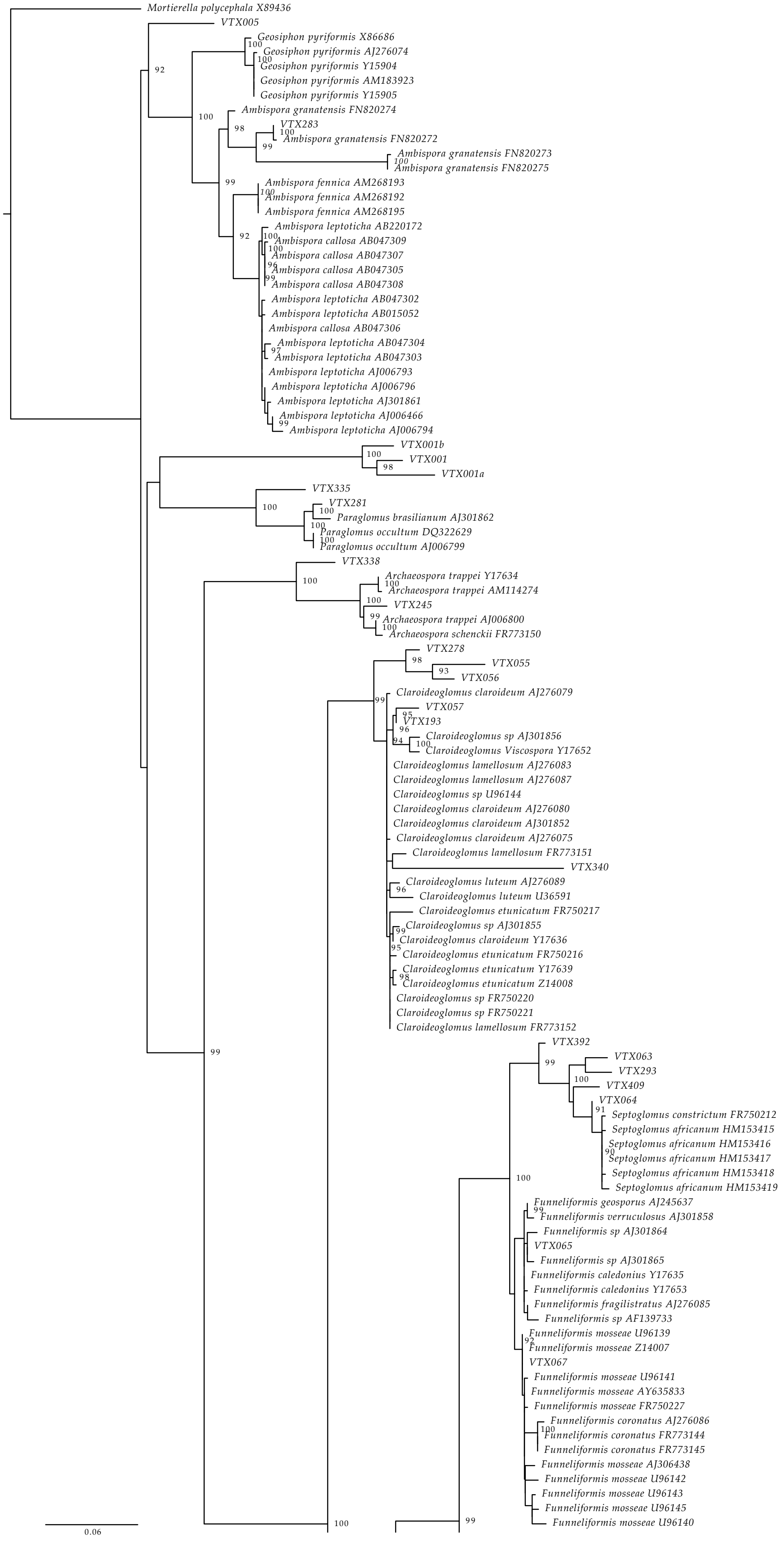

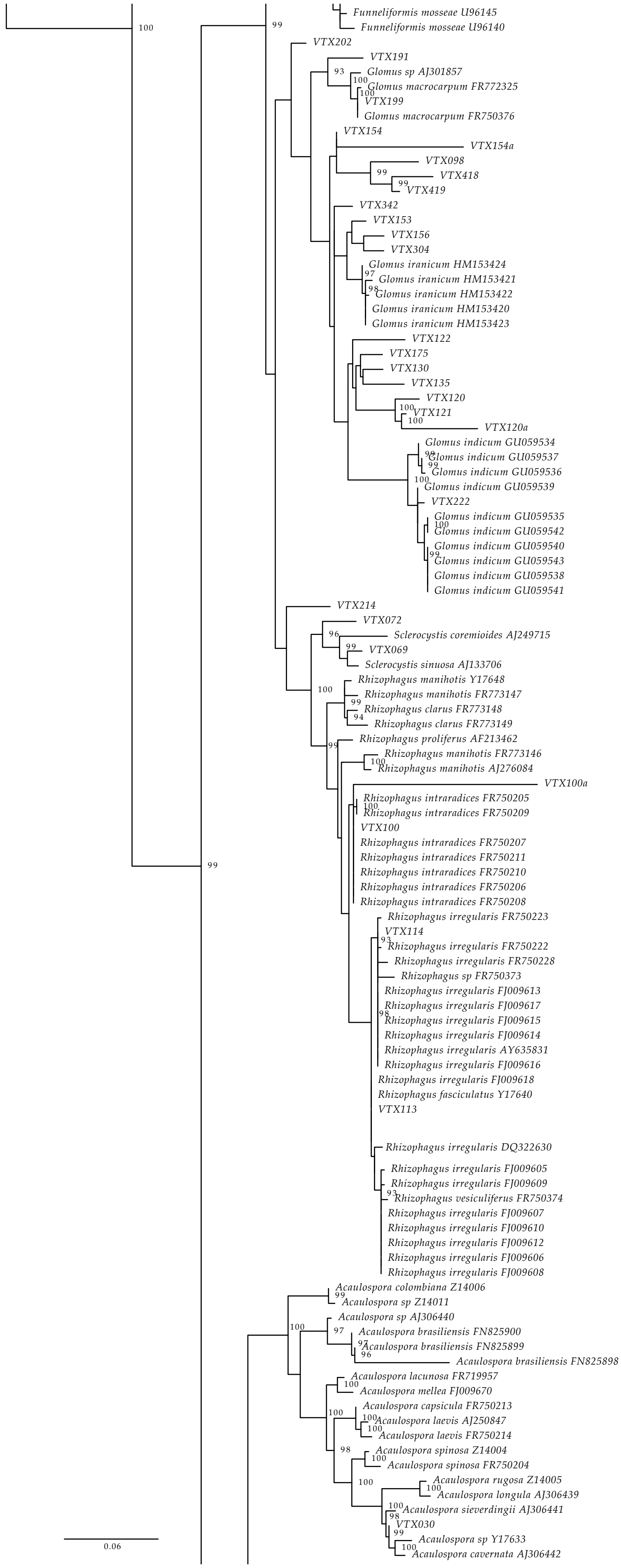
Funneliformis mosseae U96145
Funneliformis mosseae U96140
VTX202
VTX191
Glomus sp AJ301857
Glomus macrocarpum FR772325
VTX199
Glomus macrocarpum FR750376
VTX154
VTX154a
VTX098
VTX418
VTX419
VTX342
VTX153
VTX156
VTX304
Glomus iranicum HM153424
Glomus iranicum HM153421
Glomus iranicum HM153422
Glomus iranicum HM153420
Glomus iranicum HM153423
VTX122
VTX175
VTX130
VTX135
VTX120
VTX121
VTX120a
Glomus indicum GU059534
Glomus indicum GU059537
Glomus indicum GU059536
Glomus indicum GU059539
VTX222
Glomus indicum GU059535
Glomus indicum GU059542
Glomus indicum GU059540
Glomus indicum GU059543
Glomus indicum GU059538
Glomus indicum GU059541
VTX214
VTX072
Sclerocystis coremioides AJ249715
VTX069
Sclerocystis sinuosa AJ133706
Rhizophagus manihotis Y17648
Rhizophagus manihotis FR773147
Rhizophagus clarus FR773148
Rhizophagus clarus FR773149
Rhizophagus proliferus AF213462
Rhizophagus manihotis FR773146
Rhizophagus manihotis AJ276084
VTX100a
Rhizophagus intraradices FR750205
Rhizophagus intraradices FR750209
VTX100
Rhizophagus intraradices FR750207
Rhizophagus intraradices FR750211
Rhizophagus intraradices FR750210
Rhizophagus intraradices FR750206
Rhizophagus intraradices FR750208
Rhizophagus irregularis FR750223
VTX114
Rhizophagus irregularis FR750222
Rhizophagus irregularis FR750228
Rhizophagus sp FR750373
Rhizophagus irregularis FJ009613
Rhizophagus irregularis FJ009617
Rhizophagus irregularis FJ009615
Rhizophagus irregularis FJ009614
Rhizophagus irregularis AY635831
Rhizophagus irregularis FJ009616
Rhizophagus irregularis FJ009618
Rhizophagus fasciculatus Y17640
VTX113
Rhizophagus irregularis DQ322630
Rhizophagus irregularis FJ009605
Rhizophagus irregularis FJ009609
Rhizophagus vesiculiferus FR750374
Rhizophagus irregularis FJ009607
Rhizophagus irregularis FJ009610
Rhizophagus irregularis FJ009612
Rhizophagus irregularis FJ009606
Rhizophagus irregularis FJ009608
Acaulospora colombiana Z14006
Acaulospora sp Z14011
Acaulospora sp AJ306440
Acaulospora brasiliensis FN825900
Acaulospora brasiliensis FN825899
Acaulospora brasiliensis FN825898
Acaulospora lacunosa FR719957
Acaulospora mellea FJ009670
Acaulospora capsicula FR750213
Acaulospora laevis AJ250847
Acaulospora laevis FR750214
Acaulospora spinosa Z14004
Acaulospora spinosa FR750204
Acaulospora rugosa Z14005
Acaulospora longula AJ306439
Acaulospora sieverdingii AJ306441
VTX030
Acaulospora sp Y17633
Acaulospora cavernata AJ306442
0.06

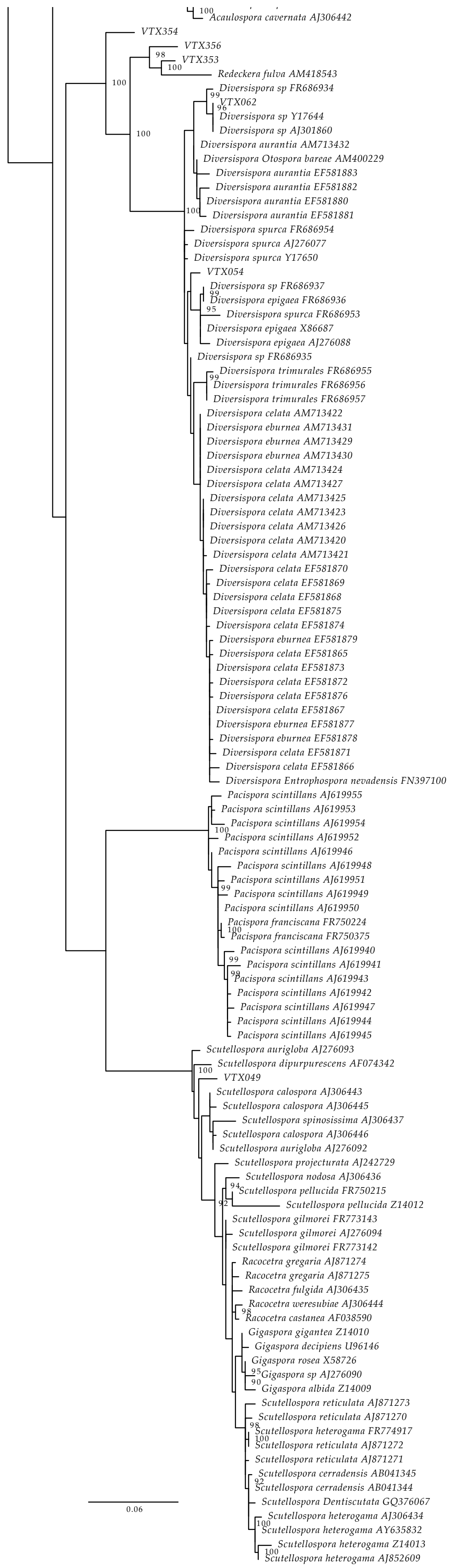
Acaulospora cavernata AJ306442
VTX354
VTX356
VTX353
Redeckera fulva AM418543
Diversispora sp FR686934
VTX062
Diversispora sp Y17644
Diversispora sp AJ301860
Diversispora aurantia AM713432
Diversispora Otospora bareae AM400229
Diversispora aurantia EF581883
Diversispora aurantia EF581882
Diversispora aurantia EF581880
Diversispora aurantia EF581881
Diversispora spurca FR686954
Diversispora spurca AJ276077
Diversispora spurca Y17650
VTX054
Diversispora sp FR686937
Diversispora epigaea FR686936
Diversispora spurca FR686953
Diversispora epigaea X86687
Diversispora epigaea AJ276088
Diversispora sp FR686935
Diversispora trimurales FR686955
Diversispora trimurales FR686956
Diversispora trimurales FR686957
Diversispora celata AM713422
Diversispora eburnea AM713431
Diversispora eburnea AM713429
Diversispora eburnea AM713430
Diversispora celata AM713424
Diversispora celata AM713427
Diversispora celata AM713425
Diversispora celata AM713423
Diversispora celata AM713426
Diversispora celata AM713420
Diversispora celata AM713421
Diversispora celata EF581870
Diversispora celata EF581869
Diversispora celata EF581868
Diversispora celata EF581875
Diversispora celata EF581874
Diversispora eburnea EF581879
Diversispora celata EF581865
Diversispora celata EF581873
Diversispora celata EF581872
Diversispora celata EF581876
Diversispora celata EF581867
Diversispora eburnea EF581877
Diversispora eburnea EF581878
Diversispora celata EF581871
Diversispora celata EF581866
Diversispora Entrophospora nevadensis FN397100
Pacispora scintillans AJ619955
Pacispora scintillans AJ619953
Pacispora scintillans AJ619954
Pacispora scintillans AJ619952
Pacispora scintillans AJ619946
Pacispora scintillans AJ619948
Pacispora scintillans AJ619951
Pacispora scintillans AJ619949
Pacispora scintillans AJ619950
Pacispora franciscana FR750224
Pacispora franciscana FR750375
Pacispora scintillans AJ619940
Pacispora scintillans AJ619941
Pacispora scintillans AJ619943
Pacispora scintillans AJ619942
Pacispora scintillans AJ619947
Pacispora scintillans AJ619944
Pacispora scintillans AJ619945
Scutellospora aurigloba AJ276093
Scutellospora dipurpurescens AF074342
VTX049
Scutellospora calospora AJ306443
Scutellospora calospora AJ306445
Scutellospora spinosissima AJ306437
Scutellospora calospora AJ306446
Scutellospora aurigloba AJ276092
Scutellospora projecturata AJ242729
Scutellospora nodosa AJ306436
Scutellospora pellucida FR750215
Scutellospora pellucida Z14012
Scutellospora gilmorei FR773143
Scutellospora gilmorei AJ276094
Scutellospora gilmorei FR773142
Racocetra gregaria AJ871274
Racocetra gregaria AJ871275
Racocetra fulgida AJ306435
Racocetra weresubiae AJ306444
Racocetra castanea AF038590
Gigaspora gigantea Z14010
Gigaspora decipiens U96146
Gigaspora rosea X58726
Gigaspora sp AJ276090
Gigaspora albida Z14009
Scutellospora reticulata AJ871273
Scutellospora reticulata AJ871270
Scutellospora heterogama FR774917
Scutellospora reticulata AJ871272
Scutellospora reticulata AJ871271
Scutellospora cerradensis AB041345
Scutellospora cerradensis AB041344
Scutellospora Dentiscutata GQ376067
Scutellospora heterogama AJ306434
Scutellospora heterogama AY635832
Scutellospora heterogama Z14013
Scutellospora heterogama AJ852609
0.06
